# Incidence and Predictors of Adolescent’s Early Sexual Debut after Three Decades of HIV Interventions in Tanzania: A Time to Debut Analysis

**DOI:** 10.1371/journal.pone.0041700

**Published:** 2012-07-27

**Authors:** Elia John Mmbaga, Frida Leonard, Germana Henry Leyna

**Affiliations:** 1 Department of Epidemiology and Biostatistics, Muhimbili University of Health and Allied Sciences, Dar es Salaam, Tanzania; 2 Pact-Tanzania, Dar es Salaam, Tanzania; Rollins School of Public Health, Emory University, United States of America

## Abstract

**Purpose:**

To determine the incidence and predictors of adolescent’s early sexual debut after three decades of HIV interventions in Tanzania.

**Methods:**

In a cross-section study of adolescents aged 16–19 residing in Morogoro Municipality, information on socio-demographic, parental-and-peer communication, and sexual behaviors were collected. Cox-regression analysis was used to examine predictors of time to sexual debut.

**Results:**

A total of 316 adolescents with mean age of 17.5±0.9 were recruited. Half (48.7%) of adolescent were sexually active with mean age at sexual debut of 14.6±2.3. Of these, 57.8% had sex before their 15^th^ birthday with incidence of early sexual debut of 17.4/1000 person-years at risk. Adolescent family characteristics, peer pressure, alcohol use, parental and peer communication were key predictors of early sexual debut.

**Conclusion:**

Parental and peer communication strategies works calling for efforts to increase its scope to reach all adolescents alongside promoting family stability and reducing adolescent alcohol consumption.

## Introduction

Since the beginning of the HIV epidemic, adolescents have been at the center of the epidemic due to their vulnerability and higher risk of infection [1]–[2]. In Tanzania, half of the population is aged less than 15 years and about 20% is between 15–24 years. The highest reported rates of sexually Transmitted Infections (STI) are found among people between 15 and 24 years; up to 60% of the new infections and half of all people living with HIV/AIDS are in this age group [3].

A recent analysis among young people provides evidence of increasing safer sexual behavior. Globally, comprehensive and correct knowledge about HIV among young people has increased slightly since 2008 but still low at an average of only 34% in Africa and 36% in Tanzania [1]. This achievement has been due to major efforts on HIV education targeting delaying of age at sexual debut, condom use and reduction of number of partners among young people [1], [4], [5], [6], [7], [8], [9].

Increasing adolescent age at sexual debut has been at the center stage of most interventions in sub-Sahara Africa [4], [7], [10], [11]. Early sexual debut increases the risk for teen pregnancy and STIs including HIV [12]. About 16 million adolescent girls aged 15–19 give birth each year, roughly 11% of all births worldwide and almost 95% of these births occur in developing countries [10]. Early age at sexual debut has been associated with higher number of lifetime sexual partners, increased risk of papilloma virus infection, cancer of the cervix and death due to abortion and teenage pregnancies. Early sex has also been attributed to higher rate of school drop-out in many parts of Tanzania with detrimental social and economic consequences.

Twenty percent of the Tanzanian population is between the ages of 15–24 and each year about three quarter of new HIV infections occur in this demographic group [13]. About 11% of adolescents below 15 years in the country have had sex and this figure increased to 13% by the year 2010 [14]. During the past three decades in Tanzania, intervention have evolved from general population approach to promote abstinence, faithfulness and condom use to high risk approaches targeting adolescents, track drivers, bar workers and STI clinic attendees. Schools have increasingly being arenas for both adolescents and parents/guardian intervention programmes addressing behavioral change communications to increase age at sexual debut and promote safer sex. These interventions have specifically targeted parental to peer, teachers to peer, and peer to peer communication using classroom teaching, drama, trained peer educators and parental counseling [6], [7], [8], [15], [16], [17]. It is therefore timely to reexamine the rate at which adolescent start sexual activities and describe determinants that guide this behavior after three decades of intervention in Tanzania. This study therefore aims at describing time to sexual debut and associated determinants among adolescents in Tanzania following three decades of HIV prevention interventions.

## Methods

### Study Design and Population

A cross sectional study was conducted in selected schools of Morogoro Municipality from March-April 2011. The study population consisted of secondary school adolescents aged 16–19 years living and studying in various secondary schools in the municipality.

### Study Area

Morogoro is a municipality in the southern highlands of Tanzania with an urban population of 206,868 [18]. The municipality is divided into nineteen wards, covering a total area of 200 square kilometers and has 26 public secondary schools. According to Demographic and Health survey data of 2010, the Eastern zone, where Morogoro municipality is located, had the highest percentage of young people aged 15–24 years old who have had sexual intercourse [14].

### Sampling and Data Collection

A multistage random sampling technique was employed in the selection of the participants in this study. A list of all public schools in the municipality was obtained from the region education department and a random sample of 6 schools out of 26 schools was selected. In each school, one form three and one form four stream (senior ordinary level classes) was randomly selected to participate in the study. Sampling from each class was done proportional to the size of the class. A self-administered structured questionnaire was administered to consenting adolescent in Swahili (the language spoken by all Tanzanian). The questionnaire collected information on socio-demographic, family and peer characteristics. Information on age at sexual debut, communication with peer and parents as well as risk behaviors was collected. All the information was collected in class under the supervision of trained research assistants in the absence of the teachers.

### Data Analysis

Data were double entered in to statistical Software for Social Scientists version 15 and later transferred in to STATA version 11.2 statistical software for analysis.

Frequencies were run for all categorical variables and means and standard deviation were calculated for continuous variables to examine sample characteristics. We examined differences between proportions using Chi square test and Student t-test was used to compare differences between means. Survival analysis was employed to examine time to age at sexual debut. Participants were considered to have failed (achieved an outcome) when they had sex at age 15 or below. This cut-off point is based on the standard UNAIDS sexual behaviors indicators. Those who never had sex at all or had sex after age 15 were considered censored. Log-rank test was used to compare the rate (incidence) of sexual debut in different groups of predictors. Considering time to event (sexual debut) nature of the analysis, Cox regression models were then built to examine sexual debut hazards ratios in different predictor variables. Different adjusted Cox regression nested models were run to adjust for various confounders and the best parsimonious model was chosen based on the log-likelihood ratio test. Due to differences between males and females with regards to sexual related behaviors and bivariate analysis of our data, we run separate analyses for male and females. All the analyses were two tailed and type-1 error rate was set at 5% level.

### Ethical Considerations

The protocol for this study was reviewed and cleared by the Muhimbili University of Health and Allied Sciences ethical committee.

The Morogoro Municipality authorities and school administrations granted permission for this study. All participants aged 18 years and above offered their written informed consents before participation. For those under 18 years, parental consents were sought and they additionally gave their written assents before participation. No names were used in the questionnaires and all the information collected were kept confidential. To reduce desirability biases, self-administered method was used and teachers were not present in the class during data collection.

## Results

### Sample Characteristics

A total of 316 adolescents from public secondary schools of Morogoro Municipality were involved in this study. Males constituted 168 (53.2%) of the respondents. Age of participants ranged from 16–19 years and the overall mean age was 17.5 [Standard deviations (SD) ±0.9] years (Males 17.4±1.0 years, versus Females 17.6±0.8, p = 0.982).

Most of the respondents reported to have obtained their primary education in urban areas 277 (87.7%). The majority of respondent’s mothers, and fathers were alive i.e. 83.2% and 75.6%, respectively, and high proportion were raised by both of their parents 186 (58.9%). Nearly half of the parents of the respondents i.e. 181 (57.3%) fathers and 157 (49.7%) mothers were reported to have completed primary level of education (Table 1).

**Table 1:** Distribution of the socio-demographic characteristics of the respondents (N = 316).

Variable	Category	n	%
Sex	Male	164	53.2
	Female	148	46.8
Age	16	51	6.1
	17	97	30.7
	18	114	36.1
	19	54	17.1
Religion	Muslim	154	48.7
	Christian	162	51.3
Grown residence	Rural	39	12.3
	Urban	277	87.7
Mother alive	Yes	263	83.2
	No	53	16.8
Father alive	Yes	239	75.6
	No	77	24.4
Raised by whom	Both parents	186	58.9
	One parent	59	18.7
	Relatives	71	22.5
Father education	Primary	157	49.7
	Secondary	106	33.5
	Post-secondary	53	16.8
Mothers education	Primary	181	57.3
	Secondary	100	31.6
	Post-secondary	35	11.1

### Incidence of Early Sexual Debut and Risk Sexual Behaviors

Of the 316 adolescent studied, 154 (48.7%) reported to be sexually active and this did not differ by sex (Males 80; 47.6% versus Females 74; 50.0%, p = 0.673). The mean age at sexual debut was 14.6±2.3 years (range 8–19 years). Mean age at sexual debut for males (14.7±2.5 years) did not differ with that of females participants (14.6±2.0), (p = 0.416). About half, (89; 57.8%) of those who were sexually active, reported to have had sex when they were aged below 15 years. The overall follow up time for all the participants was 5098 Person-Years at Risk (PYAR). This gave an incidence of early sexual debut (sex below 15 years) of 17.4/1000 PYAR. The rate of sexual debut was 18.8/1000PYAR for males and 16.2/1000PYAR for females (p = 0.324).

Seven percent (n = 11) of the sexually active group reported to have had taken alcohol the first time they had sex and 46(29.9%) also reported their partners to have taken alcohol. Half (49.7%) of the respondents reported more than two lifetime sexual partners with (13.1%) reporting more than two sexual partners six months preceding the survey. A significant proportion of female adolescents 40.3%(30) than male 17.3%(14) reported to have had sex for money (p = 0.002) (overall proportion of paid sex was 28.1%).

### Bivariate Results of Predictors of Early Sexual Debut

Table 2 present the association between incidence of early sexual debut and socio-demographic and parental/guardian predictors by sex. Having grown in urban area, having a mother alive, being raised by both parents and currently living with both parents was associated with lower incidence of early sexual debut. Having an alive father was associated with lower incidence of early sexual debut among men but not among women (p = 0.045 for male versus p = 0. 151 for females). Again, incidence of early sexual debut was significantly higher among male whose mothers had attained secondary education but not among female (p = 0.026 versus p = 0.531, respectively).

**Table 2:** Association between Incidence of sexual debut and socio-demographic and parental/guardian related predictors by sex among adolescents in Morogoro, Tanzania (N = 316).

		Males (n = 164)				Females (n = 148)			
Variable	category	Events	PYAR♯	IR†	P-value	Events	PYAR	IR	p-value
Age	16	16	543	29.4	0.052	6	207	28.9	0.661
	17	12	776	15.5		14	757	18.4	
	18	11	907	12.1		18	983	18.3	
	19	5	481	10.4		7	444	15.7	
Religion	Muslim	17	1317	12.9	0.162	23	1197	19.2	0.871
	Christian	27	1390	19.4		22	1194	18.4	
Grown residence	Rural	9	316	28.4	0.050	13	278	46.7	0.000
	Urban	35	2391	14.6		32	2113	15.1	
Mother alive	Yes	29	2258	12.8	0.000	34	2036	16.6	0.030
	No	15	449	33.4		11	355	30.9	
Father alive	Yes	29	2084	13.9	0.045	30	1802	16.6	0.151
	No	15	623	24.1		15	589	25.4	
Fathers education	Primary	23	1310	17.5	0.804	25	1210	20.6	0.441
	Secondary	14	890	15.7		12	835	14.4	
	Post-secondary	7	507	13.8		8	346	23.1	
Mothers education	Primary	34	1653	20.5	0.026	25	1240	20.1	0.531
	Secondary	6	851	7.0		12	801	15.0	
	Post-secondary	4	203	19.7		8	350	22.8	
Raising person	Both parents	14	1733	8.0	0.000	14	1366	10.0	0.000
	One parent	9	374	24.0		20	537	37.2	
	Relatives	21	600	35.0		11	488	22.5	
Current live with	Parents	18	1766	10.2	0.000	14	1469	9.5	0.000
	Relatives	20	586	34.1		19	670	28.3	
	Siblings	6	355	16.9		12	252	47.6	
Relation to	Very good	18	1606	11.2	0.006	14	1240	11.3	0.000
parents/guardian	Good	14	719	19.5		15	767	19.6	
	Average/poor	12	382	31.4		16	384	41.7	
Person easy to talk	None	6	216	27.8	0.070	7	108	64.8	0.000
to	Father	9	338	26.6		4	140	28.6	
	Mother	15	1033	14.5		23	192	17.8	
	Both	14	1120	12.5		11	851	12.9	
Talked about puberty to parent/guardian	Yes	10	984	10.2	0.038	15	1507	9.9	0.000
	No	34	1723	19.7		30	884	33.9	
Discussed	Yes	15	1173	12.8	0.183	18	1544	11.6	0.001
relationship with parent/guardian	No	29	1534	18.9		27	847	31.8	
Talked about delay	Yes	18	1758	10.2	0.000	19	1574	12.0	0.001
sex with parent/guardian	No	26	949	27.4		26	817	31.8	
Discussed impact of	Yes	16	1797	8.9	0.000	24	1878	12.8	0.000
early sex with parent/Guardian	No	28	910	30.7		21	513	40.9	
Discussed teen	Yes	16	1513	10.5	0.004	19	1683	11.3	0.000
pregnancy with parent/guardian	No	28	1194	23.4		26	708	36.7	
Discussed about	Yes	19	1562	12.2	0.027	18	1639	10.9	0.000
HIV with parent/guardian	No	25	1145	21.8		27	752	35.9	
Parent knows your	Yes	30	2258	13.3	0.002	27	1905	14.2	0.000
friends	No	14	449	31.2		18	486	37.0	
Parent drink alcohol	Yes	13	514	25.3	0.057	21	561	37.4	0.000
	No	31	2193	14.1		24	1830	13.1	

♯ PYAR, Person years at risk of sexual debut;

† IR, Incidence rate, p-value from log-rank test.

Parental factors which were associated with significant lower incidence of sexual debut at an early age were related to parental-adolescent communications. Parents/guardians who have talked or discussed with their children about puberty, relationship, delayed sex, impact of early sexual debut, teenage pregnancy and HIV had their children having lower likelihood of early sexual debut as compared to those parents who did not (Table 2). Having a very good relationship with parents was associated with lower rate of engaging in early sexual debut among both males (p = 0.006) and females (p = 0.000) adolescents. Adolescents who reported their parents to know their friends were found to have lower rate of engaging in early sexual debut as compared to those with parents who did not know their friends. Moreover, parents who did not drink impacted significantly on the rate of sexual debut of their female adolescents and borderline significant among their male adolescents (Table 2).

Adolescent engagement and relationship with peers and how this impacted early sexual debut was examined. The findings of peer related factors and their association with the rate of early sexual debut are presented in Table 3. Adolescents who reported to have ever had or currently having boy/girlfriends had higher rate of early sexual debut than those who did not.

**Table 3:** Association between Incidence of sexual debut and peer related predictors by sex among adolescents in Morogoro, Tanzania (N = 316).

		Males (n = 164)				Females (n = 148)			
Variable	category	Events	PYAR♯	IR†	p-value	Events	PYAR	IR	p-value
Have older	Yes	7	415	16.9	0.904	6	434	13.8	0.353
sibling regnant at less than 20 years	No	37	2292	16.1		39	1957	19.9	
Ever had	Yes	39	1436	27.2	0.000	38	1057	36.0	0.000
boy/girlfriend	No	5	1271	3.9		7	1334	5.2	
Currently have	Yes	29	626	46.3	0.000	32	684	46.7	0.000
a boy/girlfriend	No	15	2081	7.2		13	1707	7.6	
Boy/girlfriend occupation	Fellow student	25	535	46.7	0.627	10	214	46.7	0.874
	College student	31	79	37.9		16	318	50.3	
	Employed	2	41	48.7		8	194	41.2	
	Do girl/boyfriend	14	2052	6.8		11	1665	6.6	
Friend have sexual partners	Yes	39	1388	28.1	0.000	40	1188	33.7	0.000
	No	5	11319	3.8		5	203	4.1	
									
Friend engage	Yes	28	1041	26.9	0.000	28	757	37.0	0.000
in sex	No	16	1666	9.6		17	1634	10.4	
									
Discussed	Yes	40	2002	20.0	0.004	43	1911	22.5	0.004
relationship with friends	No	4	750	5.7		2	480	4.2	
Friend ever	Yes	23	805	28.6	0.000	23	947	24.3	0.029
	No	21	1902	11.0		22	1444	15.2	
been pregnant or made someone pregnant									
You drink	Yes	13	354	36.7	0.000	12	210	57.1	0.000
alcohol	No	31	2353	13.2		33	2181	15.1	
									
Peer drink	Yes	21	733	28.6	0.001	18	431	41.8	0.000
alcohol	No	23	1974	11.7		27	1960	13.8	

♯ PYAR, Person years at risk of sexual debut;

† IR, Incidence rate, p-value from log-rank test.

Not having a friend who has a boy/girlfriend, who engages in sex, and communication with friends regarding relationship, was associated with lower rate of early sexual debut. Alcohol consumption by the participant or by his/her peer was associated with higher incidence of early sexual debut for both sexes (p<0.001).

### Independent Predictors of Early Sexual Debut

The results of adjusted Cox regression analysis of independent predictors of early sexual debut are depicted in Tables 4 and 5. Increase in age from 18 years and above was significantly associated with decreasing rate of early sexual debut among male adolescents but not among females. Growing up in urban areas was associated 60% and 80% lower hazard rate of early sexual debut among males, and females, respectively. Higher rate of engaging in early sexual debut was reported by adolescents who did not have their mothers alive or being raised by relatives or single parent. Not having a father predicted a male adolescent higher rate of early sexual debut (Hazard Ratio (HR), 2.0, 95%CI: 1.0–3.7) but not female adolescents (HR, 1.5, 95%CI: 0.8–2.8).

**Table 4:** Adjusted Cox regression analyses of socio-demographic and parental/guardian predictors of sexual debut among adolescents in Morogoro, Tanzania (N = 316).

		Males (n = 164)		Females (n = 148)	
Variable	category	HR(95%CI)‡	P-value	HR(95%CI‡)	P-value
	16	1		1	
	17	0.5(0.2–1.1)	0.091	0.6(0.2–1.5)	0.306
	18	0.4(0.1–0.9)	0.027	0.690.2–1.5)	0.323
	19	0.3(0.1–0.9)	0.050	0.5(0.1–1.5)	0.263
Religion	Muslim	1		1	
	Christian	1.4(0.8–2.7)	0.199	0.9(0.5–1.6)	0.821
Grown residence	Rural	1		1	
	Urban	0.4(0.2–0.9)	0.040	0.2(0.1–0.5)	0.000
Mother alive	No	3.0(1.6–5.6)	0.001	2.0(1.0–5.0)	0.035
Father alive	No	2.0(1.0–3.7)	0.029	1.5(0.8–2.8)	0.185
Raised by	Both parents	1		1	
	Single parent	4.1(1.7–9.5)	0.001	3.8(1.9–7.7)	0.000
	relatives	5.7(2.9–11.4)	0.000	2.5(1.1–5.7)	0.019
Fathers education	Primary	1		1	
	Secondary	0.8(0.4–1.7)	0.724	0.6(-.3–1.3)	0.280
	Post-secondary	0.7(0.3–1.7)	0.527	1.1(0.4–2.4)	0.804
Mothers education	Primary	1		1	
	Secondary	0.3(0.1–0.8)	0.014	0.7(0.3–1.4)	0.334
	Post-secondary	0.9(0.3–2.6)	0.914	1.1(0.5–2.4)	0.776
Current live with	Parents	1		1	
	Relatives	3.4(1.8–6.6)	0.000	3.5(1.7–7.0)	0.000
	Siblings	1.9(0.7–4.8)	0.166	6.5(3.6–14.3)	0.000
Relation to parents/guardian	Very good	1		1	
	Good	1.6(0.8–3.3)	0.144	1.6(0.8–3.4)	0.173
	Average/poor	3.0(1.4–6.2)	0.003	4.4(2.1–9.3)	0.000
Person easy to talk to in the family	None	1		1	
	Father	1.2(0.4–3.6)	0.640	0.3(0.1–1.2)	0.106
	Mother	0.5(0.2–1.3)	0.161	0.2(0.08–0.4)	0.000
	Both	0.4(0.1–0.9)	0.038	0.1(0.06–0.4)	0.000
Talked about puberty to parent/guardian	No	2.0(1.0–4.1)	0.044	3.8(2.0–7.0)	0.000
Discussed relationship with parent/guardian	No	1.5(0.8–2.8)	0.198	2.9(1.6–5.4)	0.000
Talked about delay sex with parent/guardian	No	3.0(1.6–5.5)	0.000	2.6(1.4–4.7)	0.001
Discussed impact of early sex with parent/Guardian	No	3.6(1.9–6.7)	0.000	3.6(2.0–6.6)	0.000
Discussed teen pregnancy with parent/guardian	No	2.1(1.1–4.0)	0.014	3.5(1.9–6.3)	0.000
Discussed about HIV with parent/guardian	No	1.9(1.0–3.5)	0.025	3.6(2.0–6.7)	0.000
Parent knows your friends	No	2.1(1.1–4.2)	0.018	2.6(1.4–4.7)	0.002
Parent drink alcohol	No	0.5(0.2–0.9)	0.044	0.3(0.1–0.6)	0.000

‡ HR-hazard ratio, CI, Confidence Interval.

**Table 5:** Adjusted Cox regression analyses of peer related predictors of sexual debut among adolescents in Morogoro, Tanzania (N = 316).

		Males (n = 164)		Females (n = 148)	
Variable	Category	HR(95%CI)‡	P-value	HR(95%CI)‡	P-value
Have older sibling who become	Yes	1		1	
pregnant at less than 20 years	No	0.9(0.4–2.1)	0.925	1.4(0.6–3.4)	0.384
Friend ever been pregnant or	Yes	1		1	
made someone pregnant	No	0.3(0.2–0.6)	0.001	0.6(0.3–0.9)	0.025
Ever had boyfriend/girlfriend	Yes	1		1	
	No	0.1(0.05–0.3)	0.000	0.1(0.05–0.2)	0.000
Currently have a boy/girlfriend	Yes	1		1	
	No	0.1(0.06–0.2)	0.000	0.1(0.07–0.2)	0.000
Boy/girlfriend occupation	Fellow student	1		1	
	College student	0.8(0.2–2.7)	0.737	1.0(0.4–2.3)	0.903
	Employed	2.0(0.4–8.9)	0.350	0.9(0.3–2.6)	0.995
Friend have sexual partners	Yes	1		1	
	No	0.1(0.05–0.3)	0.000	0.1(0.04–0.2)	0.000
Friend engage in sex	Yes	1		1	
	No	0.3(0.1–0.6)	0.001	0.2(0.1–0.4)	0.000
Discussed relationship with	Yes	1		1	
friends	No	0.2(0.07–0.6)	0.005	0.1(0.04–0.6)	0.014
Friend ever been pregnant or made someone pregnant	Yes	1		1	
	No	0.3(0.2–0.6)	0.001	0.6(0.3–0.9)	0.021
You drink alcohol	Yes	1		1	
	No	0.3(0.1–0.6)	0.002	0.2(0.1–0.4)	0.000
Peer drink alcohol	Yes	1		1	
	No	0.3(0.2–0.7)	0.002	0.2(0.1–0.5)	0.000

‡ HR-hazard ratio; CI-Confidence Interval.

Attaining secondary education by the mother was associated with 70% reduced rate of engaging in early sexual debut among the male adolescents but this was not a significant predictor among female adolescent.

Living with relatives or having an average or poor relationship with parents was associated with higher rate of early sexual debut for both male and female adolescents. Easiness to communicate with the mother or both parents was a significant predictor of lower rate of early sexual debut among female adolescents (p = 0.000), but not among male adolescents.

As reported in the bivariate analysis, lack of parental communication with regards to puberty, relationship, delayed sex, impact of early sex, teen pregnancy, HIV and parent not knowing friends of his/her child was associated with higher hazard of early age at sexual debut. Additionally, having a parent who does not consume alcohol was associated with 50% and 70% lower likelihood of engaging in early sexual debut for both male and female adolescents, respectively.

Peer related predictors of early sexual debut are presented in Table 5. Not having a history of past or current boy/girlfriend or not having a friend who is in relationship or engages in sexual activity was associated with adolescent reduced likelihood of engaging in early sexual debut. Likewise, lack of communication with friends regarding relationship and not consuming or not having peers who consume alcohol was associated with significantly lower hazard for engaging in early sexual debut.

## Discussions

We have shown the magnitude and rate of early sexual debut to be high among adolescents in this population. Growing up residence, parental presence, parental relationship, and communication as well as peer pressure and relation had greater influence in early sexual debut among adolescents.

Age at first sex is an important indicator of exposure to risk of pregnancy and sexually transmitted infections including HIV among adolescents. As in many parts of Sub Saharan Africa sexual activities begins early in Tanzania. Previous school based studies conducted in Tanzania [19], [20] have shown the mean age at sexual debut to be 15.5 years. A school-based cluster randomized control trial among 12–14 years conducted in Dar es Salaam reported the mean age at sexual debut of 12 years at baseline, remaining almost stable over the follow up period [21]. Given the standard deviation, all these estimates are comparable to the mean age at first sex in our study which was 14.6 years.

About half of our respondent reported to have had sex and more than half of these had sex at age 15 years or below. The findings of large proportion of sexually active adolescents are similar to what had been reported in others previous studies among adolescents in Dar es Salaam [19], [20]. However, our estimate of proportion of adolescents reporting early age at sexual debut was higher than what was reported by Tanzania Demographic and Health surveywhich indicated a propotion of 6.9% and 12.8% among male and female adolescents age 15–24 years, respectively. Consistency to our findings, the proportion of young people who had early sexual debut was lower in urban areas than rural areas. Previous study in the area has also indicated a higher rate of sexual activity among adolescent in Morogoro supporting the findings of this study [13]. The location of Morogoro Municipality as an area busy with transit of cargo and passenger vehicles to the north and west of the country could partly explain the higher rates observed. This could also explain the higher rate of transactional sex of about 28% in this study as compared to the national average of 11% [14]. Given the maximum age in our sample of 19 years, the rate of sexual activity and early age at sexual debut was relatively higher than what has been reported elsewhere in Africa [22], [23].

This study found a significant association between maternal orphan hood and early sexual debut. A study in Zimbabwe by Nyamukapa et al corroborate this findings [24]. In their study, maternal orphans, regardless of gender, were more likely than non-orphans or paternal orphans to have initiated sexual activity. Orphaned adolescents may be especially vulnerable to early sexual debut through several mechanisms. For instance, orphans’ living arrangements may increase their susceptibility to early sexual activity. Single orphans, those who have lost only one parent, are less likely to live with their surviving parent, especially if they are maternal orphans [24], [25], [26], [27]. Children who were raised by relatives reported early sexual debut and this could be a results of lack of close supervision and affection that may lead to opportunities to engage in to sexual activities. Feeling of being neglected may compel adolescents to use sexual activity as a way to gain love and affection not provided at home [4], [15], [24], [27]. Increase in age and being raised in urban areas were associated with lower rate of early sexual debut in our study. This could be explained by the fact that as adolescents mature, they acquire the knowledge and skills to negotiate for sex [19]. Moreover, urban adolescents are more likely than rural raised ones to be exposed to ongoing education interventions.

Parent-child communication with regards to sexuality has been advocated by many interventions [17]. This has been so due to premise that close parenting and guardianship play important role in shaping adolescent behaviors [28]. This has often been linked to adolescent sexual attitudes and behaviors and promotion of teenagers’ discussions with their partners about sex and condom use [29]. While there is evidence that teenagers prefer to receive information about sexuality from their parents, in reality few have this privilege [30]. The same was found in this study where majority of those who reported to have poor relationship or never communicated to their parents regarding sexuality had higher incidence of early sexual debut. Lack of communication between parent/guardian and adolescent could be contributed to busy schedules parents/guardian have and the limitations set by the African culture. Tanzanian culture ascribes uncles/aunts as the main source of sexual knowledge to adolescents. This arrangement was possible in the old extended family environment, but as family became less extended especially in urban setting, parents/guardian needs to take over this responsibility. Therefore, current interventions should be geared towards improving parental communication skills and increasing and improving better interaction between parents/guardians and adolescents.

As reported in other studies, presence of the mother had higher impact on adolescent rate of engaging in early sexual debut especially for female adolescents. Father impacted significantly on the reduction of early sexual debut among male than women adolescents. The preference of female adolescents to their mothers and male adolescents to their fathers has been reported in Tanzania [16]. Presence of further figure regardless of communication has impact on male adolescent’s sexual behaviors than females.

In this study, we found peer influence and state of communication to be a strong predictor of early sexual debut. Being in sexual relationship and alcohol use among peer and by the respondents were associated with higher rate of early sexual debut. Reviews of previous research highlight aspects of adolescents’ friendships that are key influences on their sexual risk behaviors. Friends’ sexual behaviors, adolescents’ perceptions of friends’ behaviors and level of involvement with friends are among the key determinants of sexual behaviors [26], [31], [32]. Perceptions of gaining the respect of friends, acceptance and curiosity of doing what others are doing can impair adolescent’s good judgment and fuel risk taking behaviors. Moreover, alcohol consumption has been reported to diminish risk perception and judgment causing not only engaging in sexual activities but also other risk behaviors [19], [33], [34]. These findings indicate the importance of parental/guardian in examining the characteristic of their children’s friends as an indicator of their children’s behaviors. Most interventions in tanzaia since the start of the HIV epidemic three dacades ago have used school as an intervention arena. This has not been very successiful in reaching a large number of parents/guardians. Modification of these approach to putting emphasis in involving parental association in schools could be improve skills building and coverage. This would also give an opportunity to convey a message on the need for cultural changes that hinder parental nvolvement in adolescets sexual education. Additionally, this paper presents evidence on the importance of family structure in shaping adolescents behaviors. It is clear that presence of both parents is instrumental in adolescent behaviors modification calling for not only communication training but also maintenance of strong family structures.

Risk sexual behaviors are often affected by desirability bias and this could affect the interpretation of the findings of this study. However, the self-administered nature of the survey, anonymity and absence of teachers in the class might have reduced this bias. We acknowledge the fact that risk and exposure vary with time and this may have an impact on the analytical assumption adopted. On the other hand, the robust nature of approach lies in the global consideration of the time to sexual debut rather than only the point of debut.

Sexual behaviors such as age of sexual debut, occupation of the first partner and number of sexual partners may be affected by recall bias. Given the recent nature of age at sexual debut of the study participants, this bias may not have influenced the validity of the estimates. Moreover, this was a school based study and did not include the out of school adolescents who might be at higher risk of early sexual debut or dropped school as consequences of early pregnancies. Therefore, although the estimates presented in this study were higher, the true general population estimate would be even higher cementing further the recommendations given in this paper.

In conclusion, this study indicate that a large proportion of Tanzanian adolescent engage in sexual activity at an early age and continue to practice risk sexual behaviors three decade after continued HIV prevention interventions. The high rate of early age at sexual debut was determined by adolescent living condition, family stability and communication with parents. Peer influence played a major role in adolescent sexual behaviors. We provide evidence that intervention measures may have improved the quality of communication between parents and peers but the scale or the coverage of communication may not be adequate. Strategies to encourage communication practices by parents against prevailing traditions should be a priority. Family stability with both parents and avoidance of bad influence peers are areas to be targeted by new and present interventions.
